# A Novel Method to Quantify Longitudinal Orthodontic Bone Changes with In Vivo Micro-CT Data

**DOI:** 10.1155/2018/1651097

**Published:** 2018-10-01

**Authors:** Chao Wang, Li Cao, Chongshi Yang, Yubo Fan

**Affiliations:** ^1^Assistant Professor, College of Stomatology, Chongqing Medical University, Chongqing, China; ^2^Chongqing Municipal Key Laboratory of Oral Biomedical Engineering of Higher Education, Chongqing, China; ^3^Attending Doctor, Department of Orthodontics, Stomatological Hospital of Chongqing Medical University, Chongqing, China; ^4^Attending Doctor & Lecturer, Department of Orthodontics, Stomatological Hospital of Chongqing Medical University, Chongqing, China; ^5^Chongqing Key Laboratory for Oral Diseases and Biomedical Sciences, Chongqing, China; ^6^Professor, Laboratory for Biomechanics and Mechanobiology of Ministry of Education, School of Biological Science and Medical Engineering, Beihang University, Beijing, China

## Abstract

Orthodontic tooth movement (OTM) is the result of region-specific bone modeling under a load. Quantification of this change in the alveolar bone around a tooth is a basic requirement to understand the mechanism of orthodontics. The purpose of this study was to quantify subregional alveolar bone changes during orthodontic tooth movement with a novel method. In this study, 12 Sprague-Dawley (SD) rats were used as an orthodontic model, and one side of the first upper molar was used to simulate OTM. The alveolar bone around the mesial root was reconstructed from in vivo micro-CT images and separated from other parts of the alveolar bone with two semicylinder filters. The amount and rate of OTM, bone mineral density (BMD), and bone volume (BV) around the root were calculated and compared at 5 time points. The results showed that the amount of tooth movement, BMD, and BV can be evaluated dynamically with this method. The molar moved fastest during the first 3 days, and the rate decreased after day 14. BMD decreased from day 0 to day 14 and returned from day 14 to day 28. BV deceased from day 0 to day 7 and from day 14 to day 28. The method created in this study can be used to accurately quantify dynamic alveolar bone changes during OTM.

## 1. Introduction

Orthodontic tooth movement (OTM) results from the modeling and remodeling of the alveolar bone under a prolonged and moderate load. With selective bone removal and apposition, the tooth moves through the alveolar bone, carrying its attachment apparatus with it. Then, the newly generated bone is remodeled to increase its bone density and mechanical strength to perform function in the new position [1].

Alveolar bone modeling has region-specific characteristics. Different processes of bone modeling can be observed at different sides under the same orthodontic load. The pressure side and tension side show bone resorption mediated by osteoclasts and bone apposition mediated by osteoblasts, respectively [1, 2]. Therefore, partitioning the alveolar bone around the root is necessary when evaluating the amount and characteristics of bone changes in orthodontics.

Bone morphologic measures, such as bone mineral density (BMD), bone volume (BV), and other microstructure parameters, were calculated by definition of regions of interest (ROIs) in previous studies [3–5]. In these studies, a cube of the alveolar bone near the root was extracted as a ROI to evaluate bone resorption and apposition. Although this method is straightforward, the calculation is affected by the relatively arbitrary nature of ROI selection, especially in in vivo studies. Therefore, determining the same ROI at different time points is important to rule out this influence.

In this study, sequential images of the Sprague-Dawley (SD) rats were acquired from in vivo micro-CT. The alveolar bone around the mesial root was reconstructed and separated from other parts of the alveolar bone with two semicylinder filters, which were defined as the ROI. BMD and BV were calculated and compared at different time points. The objective of this study was to accurately quantify dynamic bone resorption and apposition with this novel method and to evaluate the modeling of the alveolar bone on tooth movement.

## 2. Materials and Methods

### 2.1. Animal Study and Micro-CT Scan

**Figure 1:**
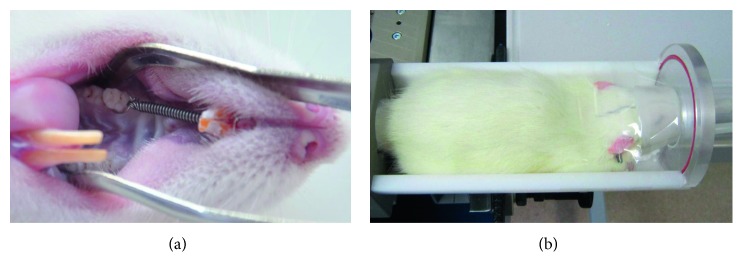
Animal study: (a) OTM of the SD rat; (b) fix and inhalation anesthesia.

This project was conducted with approval from the Ethics Committee of Chongqing Medical University. Twelve male SD rats (aged 6–8 weeks and weighing 180–220 g) were used as the experimental animals and were fed a standardized laboratory rat diet under conventional conditions (25 ± 2°C and a 12-hour light/dark cycle). The right maxillary first molar of each rat served as the orthodontic side, and the left first molar served as a control. On the orthodontic side, the first molar and incisor were connected with a nickel-titanium coil spring (wire diameter: 0.008 mm; Protect, Zhejiang, China). The coil spring was activated to generate a about 25 g continuous force to move the first molar forward (Figure 1(a)). The maxillary left first molar served as the control without any orthodontic load. The animal study protocol has been previously described [6].

Each animal was scanned with an in vivo micro-CT (viva CT40, SCANCO Medical, Brüttisellen, Switzerland) under isoflurane anesthesia (2.3–2.5 v/v %) at day 0 (before the orthodontic load) and days 3, 7, 14, and 28 after orthodontic loads (Figure 1(b)). The scan settings were 70 kV, 114 *μ*A, and 350 ms integration time with an isotropic voxel resolution of 10.5 *μ*m and a calibration with 1200 mg HA/ccm. The scan started 3 mm before the mesial root of the first molar and included the entire first molar and the surrounding alveolar bone. The scan field was determined in 2D X-ray scout view. The coil springs were removed before scanning and reattached after scanning to prevent metal artifacts. In this study, each scan lasted 45 min, and approximately 600 cross-sectional images were generated. The rats were sacrificed by overinhalation of CO_2_ after the last scan. These images acquired from scanning were exported in the Digital Imaging and Communications in Medicine (DICOM) format and processed by a graphic workstation.

### 2.2. Image Processing: Measurement of OTM Distance

**Figure 2:**
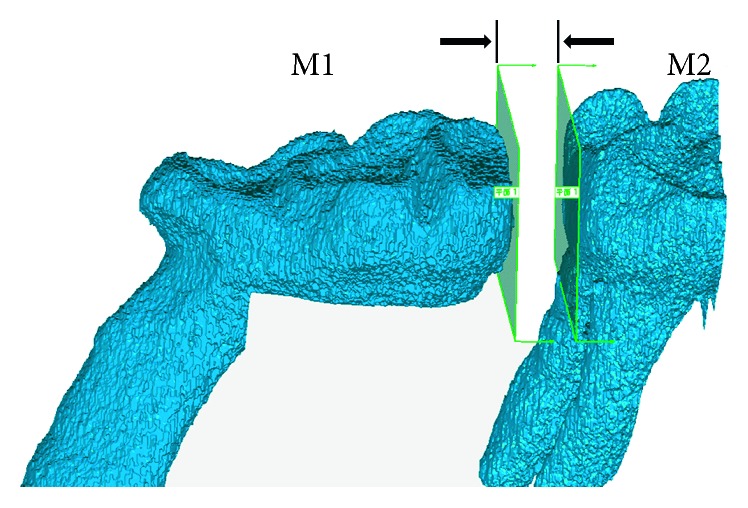
Measurement of OTM distance.

The measurement of tooth movement was calculated as the distance between the proximal surfaces of the first and second molar. The images generated from micro-CT were imported into Mimics software (version 10.0, Materialise, Leuven, Belgium) to segment the crowns of the first and second molars in 2D sections by determining the Hounsfield value and manual mask segmentation, such as region growth, morphology, and edit operations. Three-dimensional objects of the crown were calculated from 2D image sequences and exported in the stereolithography (STL) format into Geomagic Studio (2012, Raindrop Inc., Rock Hill, South Carolina, USA). A plane was fitted according to the shape of the distal surface of the crown of the first molar and transferred to the second molar until tangent to the mesial surface of the second molar. The distance of the plane between the two positions can be determined as the tooth movement distance (Figure 2). The amount of tooth movement at 5 time points can be calculated with this method.

### 2.3. Image Processing: the Calculation of BV and BMD

The first molar of the SD rats has five roots; the mesial root is the largest and is far from the other four roots, thus enabling clear evaluation of the modeling of the alveolar process around the mesial root.

In this study, a novel method was created to quantify alveolar bone changes under an orthodontic load through the following steps: 3D reconstruction of the tooth, superimposition of 3D images at different time points, extraction of the alveolar bone around the root, and calculation of parameters in SCACO.

#### 2.3.1. Reconstruction of the Alveolar Bone, Crown, and Mesial Root of the Upper First Molar

**Figure 3:**
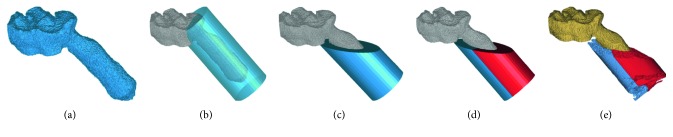
The reconstruction, partition, and extraction of the alveolar bone around the mesial root: (a) the reconstruction of the crown and mesial root of the upper first molar; (b) generation of the cylinder around the root; (c) partition of the cylinder with the alveolar crest plane; (d) division of the cylinder into the mesial and distal sides according to the direction of the orthodontic load; (e) reconstruction of the alveolar bone within semicylinder filters.

The crown and mesial root of the first molar around the alveolar bone were reconstructed and exported in the STL format with the abovementioned method (Figure 3(a)).

#### 2.3.2. Formation of Cylinder Filters at Different Time Points

3D images of the molar and alveolar bone were imported into Geomagic Studio to generate a cylinder that can be used to define the ambit of the quantified bone. The height of the cylinder was determined by the following points: the start point was the intersecting point of the occlusal surface and the long axis of the mesial root; the end point was 0.4 mm past the apical according to the anatomic characteristics at this region. The diameter was set as 1.8 mm, taking into consideration the distance to the other roots (Figure 3(b)). The cylinder was converted into a CAD object and partitioned with the plane of the alveolar bone ridge (Figure 3(c)). Then, the cylinder was divided into the mesial part and distal part according to the direction of the orthodontic load (Figure 3(d)).

There were five image sequences corresponding to 5 scans for each animal. The positions of the upper first molar and surrounding structures varied at different time points as the rat cannot be fixed in the exact same position during scanning. Therefore, the image at one time point needs to be matched to the others through rotating and translating operations. In this study, the initial scan (day 0) was set as the baseline and superimposed onto the latter images (days 3, 7, 14, and 28) with its semicylinders. The buccal and lingual grooves on the crown were chosen as references of superimposition as these characteristics are obvious, easily recognized, and not subjected to abrasion caused by mastication. The cylinder generated at time point 1 was then allocated to the roots at other time points with the same size. The CAD objects of the two semicylinders were generated and saved in the STL format.

#### 2.3.3. Alveolar Bone Extraction around the Mesial Root

The STL objects were projected to 2D sections generated by micro-CT scans by calculating the mask from an object operation. The alveolar bone within semicylinders was extracted to form a new mask through a Boolean operation and exported in the DICOM format (Figure 3(e)).

#### 2.3.4. Analysis in SCANCO Analysis Module

**Figure 4:**
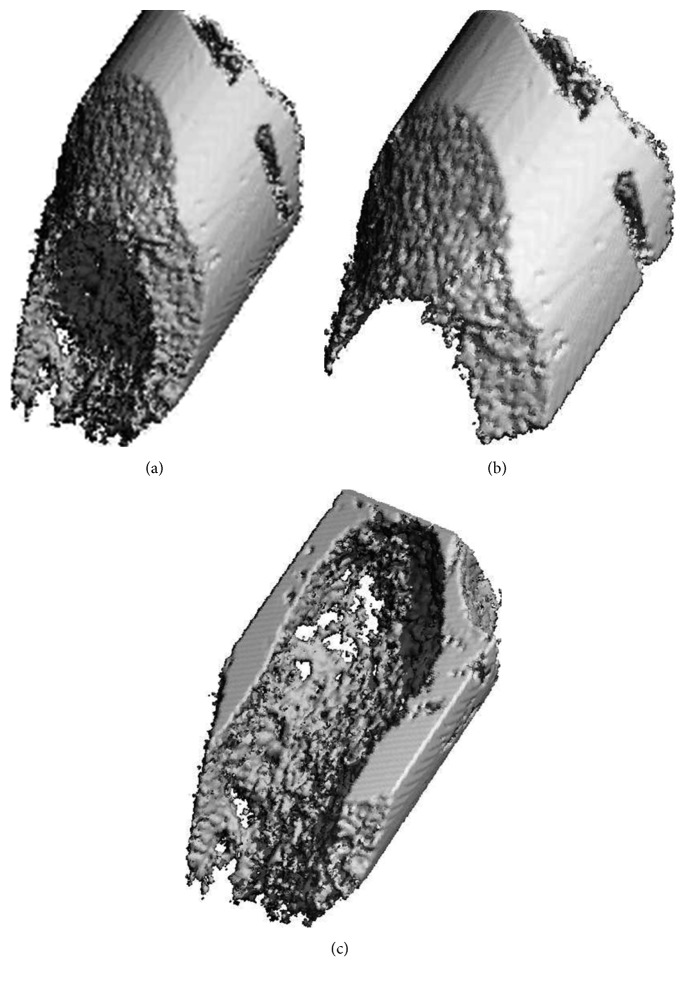
Reconstruction of the extracted alveolar bone around the mesial root (a), and mesial part (b) and distal part (c) in SCANCO viva CT40.

The DICOM files generated in the previous step were imported into the evaluation module of SCANCO viva CT40 (version 4.1). Bone modeling parameters, such as BMD and BV, were calculated at different time points after determination of the ROI, which included all the images within semicylinders and were rendered with a value of 3–5 HU (the images have reached the threshold in Mimics software and were used to generate dichromatic images: background < 3 HU, alveolar bone > 3 HU) (Figures 4(a)–4(c)).

### 2.4. Statistics

Statistical analysis was performed using SPSS software (version 13.0, SPSS Inc., Chicago, USA). Repeated measures analysis of variance was used for comparisons of the rate of tooth movement and bone morphology parameters, including BMD and BV, among different time points.

## 3. Results

**Figure 5:**
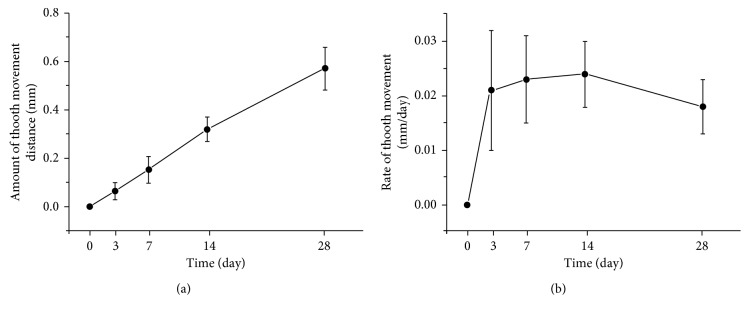
Amount and rate of tooth movement on the orthodontic side: (a) amount of orthodontic tooth movement; (b) rate of orthodontic tooth movement.

The upper first molars of the SD rats on the orthodontic side moved mesial under the load of the Ni-Ti spring (Figures 5(a) and 5(b)). The blank control side showed no tooth movement. The distance at adjacent time points significantly increased (*P*<0.05). The amount of OTM steadily increased over time within the first 2 weeks and slightly slowed down over the last 2 weeks (Figure 5(a)). The rate of OTM rapidly increased during the first 3 days (*P*<0.05), slightly increased from day 3 to day 14, and slightly decreased from day 14 to day 28 (Figure 5(b)).

**Figure 6:**
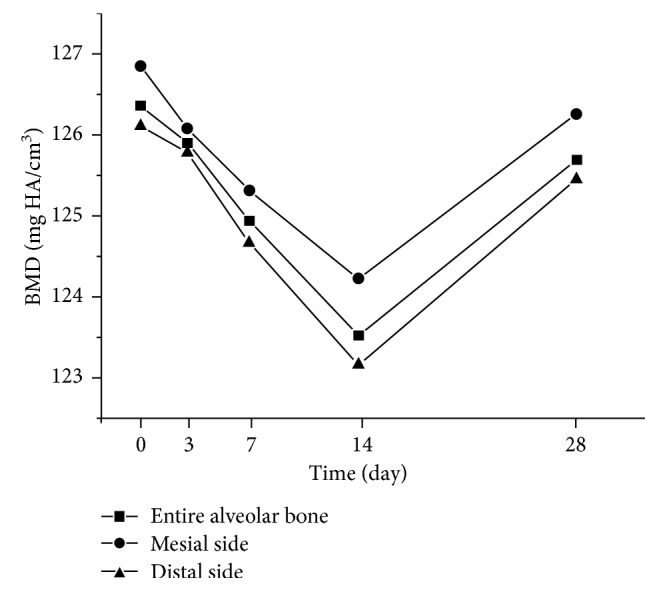
BMD changes in the alveolar bone at different time points.

Figure 6 shows the BMD changes in the alveolar bone around the mesial root at different time points. The BMD value significantly changed between adjacent time points (*P*<0.05); BMD decreased at day 3, reached its lowest point at day 14, and then markedly returned. The mesial and distal parts of the alveolar bone showed the same changes as the entire bone. However, the BMD of the alveolar bone on the mesial side was higher than that of the entire bone, and the BMD of the distal part was lower than that of the entire bone.

**Figure 7:**
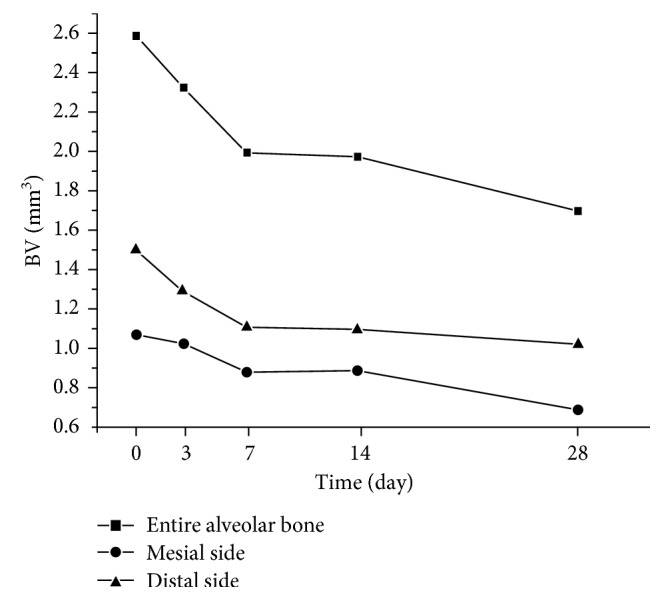
BV changes in the alveolar bone at different time points.

The volume of the alveolar bone around the mesial root is the sum of its mesial and distal parts. The three parts showed the same change tendency, decreasing from the initial time point to day 28 (Figure 7). The decrease in BV at the mesial part was more obvious than that at the distal part from day 14 to day 28 (*P*<0.05).

## 4. Discussion

Direct measurement of tooth movement distance in the rat specimens is infeasible due to the smaller molar size (the length of the crown is approximately 3 mm), the tiny initial tooth movement (<0.5 mm), and insufficient exposure of the distal space for the posterior location of the molar. Presently, the main approach is 2D measurement by determining the distance of the landmarks on the molars with X-ray films [7, 8]. However, this method suffers from more errors caused by inconsistent X-ray magnification, projection angulation, and other factors, such as interference from artifacts caused by other anatomic structures. Currently, micro-CT is used to measure tooth movement distance in rodents [3, 9]. More precise linear and angulation measurements can be acquired in three-dimensional micro-CT films, which display the teeth and surrounding apparatus without the interference of other structures. However, the nature of this approach is a 2D measurement with greater arbitrariness when determining the direction of tooth movement, especially when the direction of tooth movement does not conform to the scan plane or vertical scan plane, which may cause greater errors. In this study, the tooth movement distance was determined by transferring a plane that was fitted according to the shape of the distal plane of the first molar, which was reconstructed from micro-CT images, to the distal plane of the second molar. This method can obtain better and more accurate tooth movement results based on micro-CT, excluding the interference of the animal position during scanning and artifacts caused by other structures.

Although OTM is a continuous process, it can be divided into the following stages: the initial stage, lag stage, and linear movement stage [10]. Tooth movement at the initial stage is caused by deformation of the PDL within 1-2 days; the lag stage shows slow tooth movement, which is blocked by hyalinization on the pressure side of the PDL and adjacent alveolar bone; and tooth movement at the linear movement stage is the fastest as the hyalinization is absorbed. The three-stage theory can clarify and explain OTM in the clinic. In this study, we did not observe marked, staged tooth movement. The amount of OTM steadily increased over time within the first 2 weeks and slightly slowed down in the last 2 weeks. The most rapid tooth movement was observed during the initial 3 days; the same level was approximately maintained after this point, as shown in Figure 5(b). Von Böhl et al. [11, 12] demonstrated that the hyalinization caused by ischemic injury to the PDL will hinder tooth movement. The nontypical tooth movement without remarkable delayed and linear movement stages observed in this study may be caused by the low possibility of hyalinization in the PDL of the rats, which have a wider PDL relative to the size of the tooth compared with that of human patients.

Bone density changes with generation and functional regulation of osteoblasts and osteoclasts. Osteoclast absorbs the surface of the alveolar bone to form an acidic microenvironment, leading to demineralization of the adjacent bone, with degradation of the collagen matrix after secretion of various hydrolases. Osteoblasts mediate bone formation through secretion of collagen and glycoprotein to form the osteoid and matrix vesicle on the osteoid to form the mineralization center. Although the mechanisms of osteoblasts and osteoclasts can be revealed in cell biology and molecular biology in histological studies, micro-CT scans can quantify their functional activity through analysis of BV and bone density. In this study, the BMD markedly decreased from the initial time point to day 14 and almost returned to the original level in the last 2 weeks (Figure 6). This result demonstrated that the entire alveolar bone modeling was dominated by bone resorption in the early stage and bone apposition in the later stage. As shown in Figure 6, the change tendency of BMD was almost the same on the mesial and distal sides. This result indicated that it cannot be considered simply that only bone resorption exits in the moving side and bone formation in the opposite side. More subregions of the alveolar bone, such as the cervix of the root or apex of the root, are needed to reflect the characteristic change of alveolar bone modeling.

The rate of OTM has a close relationship with bone density. Hashimoto et al. found that the rate of OTM increased remarkably with a decrease in bone mineral content (BMC) after ovariectomy in the Wistar rats [3, 13]. Conversely, OTM decreased with an increase in bone minerals after diphosphonate treatment [14–16]. These findings indicate that the rate of OTM is negatively correlated with bone mineral content. Our study verified this result. The molar showed the largest velocity when the bone density on the pressure side was the lowest and then exhibited slower OTM when bone density increased.

Bone loss caused by orthodontic treatment seems inevitable, especially when tooth movement occurs at the extraction space and lower incisors [17, 18]. The most common method to assess bone loss in orthodontic treatment is distance measurement from 2D section films reconstructed from CBCT [17–19]. Bone resorption can also be detected and evaluated with the BV parameter three-dimensionally. In this study, the entire alveolar bone around the mesial root decreased at the initial stage and final stage, and the decrease on the mesial side was more obvious after 14 days (Figure 7). This decrease is not only the result of alveolar bone resorption caused by OTM but also the result of the reduction in the alveolar ridge height when the tooth moved forward.

Traditionally, bone modeling and remodeling can be evaluated through osteoclast counts, visualization of absorption lacuna, and new bone apposition from histologic films or semiquantification of cytokines, such as PCNA and TGF beta-1, from immunohistochemical staining of sections [20]. Another option is quantification of RNA markers of osteoclastic cells and osteoclastic regulators, such as M-CSF, RANKL, OPG, OPN, BSP, and OCN [7]. These methods are more sensitive in detecting bone changes. However, these methods are invasive and have to be done via the tissue biopsy or autopsy after animals are sacrificed; thus, more experimental animals are needed in a longitudinal study. The micro-CT technique as a noninvasive method is more suitable for observing and quantifying bone changes in the same location over a longer period of time. Another advantage of micro-CT is volume measurement with a wider scope versus a slice or thin layer with histological methods. Therefore, micro-CT and imaging process were utilized to quantify bone changes of subregional bone segments in this study.

In vivo micro-CT offers the possibility to monitor dynamic changes in the bone microstructure through repeated scanning. However, studies about the effect of repeated radiation from micro-CT on small animals are lacking. Several studies have illustrated that repeated radiation with lower doses of 400∼600 mGy is unlikely to have an impact on any changes in bone microstructures [21]. However, more extensive research is still needed to clarify the effect of radiation on bone modeling and remodeling according to different scan protocols. Waarsing et al. [22] first quantified dynamic changes in the microstructure of the rat tibia after ovariotomy based on image registration of in vivo micro-CT films. Schulte et al. demonstrated the sensitivity and repeatability of bone formation and bone resorption characteristics, such as mineral surface (MS), mineral apposition rate (MAR), eroded surface (ES), and mineral resorption rate (MRR), acquired with in vivo micro-CT scans compared with those acquired with fluorescent staining [23]. Nishiyama et al., Liu et al., and Lukas et al. [24–26] demonstrated the broad application prospects in experiments. Based on these studies, we created a method to quantify alveolar bone changes through image processing based on in vivo micro-CT scans.

The main difference between the method in this study and the traditional method is the definition of ROI. In the traditional method, the ROI cannot be accurately located in the same place due to the position change of anatomic structures caused by the inability to maintain the same animal positions during several scans. Another disadvantage is the inflexible definition of the border and shape of the ROI, which should be defined according to research objectives and anatomic characteristics. By contrast, in this study, the ROIs were all the same and related to the root and crown in several scans. Moreover, the partition can be flexibly performed according to research demands, such as the direction of the orthodontic load. However, the determination and registration of ROI may increase the labor intensity. Moreover, high resolution images are needed to construct the anatomic structures used for registration at different time points.

Furthermore, with the strategy and technical route created in this study and the wide use of CBCT, we can obtain a more complicated, accurate, and flexible evaluation of the bone remodeling and modeling effects in the clinic treatments, such as space-closing, expansion, maxillary protraction, functional treatment, and corticotomy.

## 5. Conclusions

The present study introduced a nondestructive 3D technique used to evaluate longitudinal bone morphology changes that result from an orthodontic load based on micro-CT images. Special regions of the alveolar bone can be extracted through image processing and partitioned according to clinical or research requirements. In addition, rat bone modeling was evaluated with this technique. The results indicated that the rate of OTM is negatively correlated with BMD. The BV of the alveolar bone decreased during the OTM procedure, and some differences were detected between the mesial and distal sides of the alveolar bone around the mesial root of the upper first molar of the SD rats.

## Data Availability

The data used to support the findings of this study are an intact data chain including original micro-CT scan images, the generated CAD files, the STLs, and DICOM files, as well as the final results calculated in in-vivo micro-CT. These files are so large (more than 250 GB) to be accessed in the network storage system. These files are available from the corresponding author upon request.
